# Association of Online Risk Factors With Subsequent Youth Suicide-Related Behaviors in the US

**DOI:** 10.1001/jamanetworkopen.2021.25860

**Published:** 2021-09-20

**Authors:** Steven A. Sumner, Brock Ferguson, Brian Bason, Jacob Dink, Ellen Yard, Marci Hertz, Brandon Hilkert, Kristin Holland, Melissa Mercado-Crespo, Shichao Tang, Christopher M. Jones

**Affiliations:** 1 National Center for Injury Prevention and Control, Centers for Disease Control and Prevention, Atlanta, Georgia; 2 Bark, Atlanta, Georgia; 3 Division of Injury Prevention, Centers for Disease Control and Prevention, Atlanta, Georgia; 4 Division of Adolescent and School Health, Centers for Disease Control and Prevention, Atlanta, Georgia; 5 Division of Overdose Prevention, Centers for Disease Control and Prevention, Atlanta, Georgia; 6 Division of Violence Prevention, Centers for Disease Control and Prevention, Atlanta, Georgia

## Abstract

**Importance:**

The association between online activities and youth suicide is an important issue for parents, clinicians, and policy makers. However, most information exploring potential associations is drawn from survey data and mainly focuses on risk related to overall screen time.

**Objective:**

To evaluate the association between a variety of online risk factors and youth suicide-related behavior using real-world online activity data.

**Design, Setting, and Participants:**

A matched case-control study was conducted from July 27, 2019, to May 26, 2020, with the sample drawn from more than 2600 US schools participating in an online safety monitoring program via the Bark online safety tool. For 227 youths having a severe suicide/self-harm alert requiring notification of school administrators, cases were matched 1:5 to 1135 controls on location, the amount of follow-up time, and general volume of online activity.

**Exposures:**

Eight potential online risk factors (cyberbullying, violence, drug-related, hate speech, profanity, sexual content, depression, and low-severity self-harm) through assessment of text, image, and video data.

**Main Outcomes and Measures:**

Severe suicide/self-harm alert requiring notification of school administrators; severe suicide alerts are statements by youths indicating imminent or recent suicide attempts and/or self-harm.

**Results:**

The 1362 participants had a mean (SD) age of 13.3 (2.41) years; 699 (51.3%) were male. All 8 online risk factors studied exhibited differences between case and control populations and were significantly associated with subsequent severe suicide/self-harm alerts when examining total direct and indirect pathways. These associations ranged from an adjusted odds ratio (aOR) of 1.17 (95% CI, 1.09-1.26) for drug-related content to an aOR of 1.82 (95% CI, 1.63-2.03) for depression-related content. When considering the total number of different types of online risk factors among the 8 measured, there was an exponentially larger risk of severe suicide/self-harm alerts; youths with 5 or more of the 8 risk factors present in their online activity had a more than 70-fold increased odds of subsequently having a severe suicide/self-harm alert (aOR, 78.64; 95% CI, 34.39-179.84).

**Conclusions and Relevance:**

The findings of this study suggest that many discrete types of risk factors are identifiable from online data and associated with subsequent youth suicide-related behavior. Although each risk factor carries a specific association with suicide-related behavior, the greatest risk is evident for youths demonstrating multiple types of online risk factors.

## Key Points

**Question:**

Are online risk factors measured using real-world online activity data associated with youth suicide-related behavior?

**Findings:**

In this case-control study of 227 youths, having a severe suicide/self-harm alert in a school-based online safety monitoring program was associated with multiple online risk factors (including cyberbullying, violence, drug-related content, hate speech, profanity, sexual content, depression, and low-severity self-harm content). However, the greatest risk was found among youth having multiple types of online risk factors.

**Meaning:**

This case-control study provides information that may help guide youth suicide prevention activities related to online risk factors.

## Teaser

This case-control study examines the association between online risk factors and suicide-related behaviors in youths using a school-based online safety monitoring program.

## Introduction

Suicide among children and adolescents is a devastating event for families and communities. Suicide is the second leading cause of death among youths aged 10 to 18 years in the US, with rates increasing 61.8% from 1999 to 2018.^1^ Rates of nonfatal suicide attempts and self-harm among youths have also increased.^1,2,3^ From 2001 to 2018, self-harm emergency department visits increased 88.6% among youths aged 10 to 18 years.^1^ Furthermore, survey data from US high school students in 2019 revealed that 18.8% of students reported having seriously considered suicide attempts.^4^ There is no single cause of suicide; rather, epidemiologic research has identified a wide range of risk factors. Leading risk factors include depression, violence victimization and perpetration, substance use, and exposure to adverse childhood experiences, such as sexual violence.^5,6,7^

School-based efforts have long been a central component of youth suicide prevention.^8^ School-based suicide prevention activities range from social-emotional learning strategies implemented among all students that teach skills that can buffer suicide risk, such as problem-solving, emotional regulation, and coping skills, to specific health education curricula, to more tailored interventions, such as group or individual counseling and behavioral health care.^7,8,9,10,11^

Risk of suicide is not always identified before a suicide attempt.^12^ Furthermore, many youth suicide attempts can be impulsive acts, challenging even the most intensive prevention efforts.^13^ Given that current suicide screening approaches are based on self-report, detection and assistance of youths at risk of suicide depends first on accurate disclosure of suicidal thoughts, which can be underreported by youths.^13,14^ As a response to these challenges and increasing rates of youth suicide, schools are increasingly examining and using digital tools to improve suicide prevention activities and identify risk as early as possible.^15^

A growing body of research has examined the importance of attention to online communications about suicide.^16,17,18^ Research from youth suicide clusters in the US has revealed that robust conversations about suicide occur among youths online and may have an important role in suicide prevention activities.^19^ The largest body of research to date examining youth suicide risk and online activities has focused broadly on risks associated with excess screen time,^20,21,22^ which is one among multiple potential suicide-related risk factors.

Assessment of screen-time exposure has been conducted via surveys such as the Youth Risk Behavior Surveillance System, administered by the Centers for Disease Control and Prevention.^23^ Although such data have provided important foundations for exploring potential associations between online exposures and youth mental health, the cross-sectional nature of these data has limited the ability to fully understand youth suicide risk. In addition, such data are self-reported, which may introduce misclassification, social desirability, or recall biases, and generally do not allow for a detailed examination of different types of online activities. This limitation is important because some online activities may increase the risk of suicide and others that provide positive social support may be helpful or confer protection against suicide.^24,25^ To date, the most rigorous longitudinal studies focusing on screen time and youth mental health have similarly used self-reported data on online behaviors to assess risk and generally can assess youths only at predefined intervals, such as annual evaluations.^26^ Thus, ongoing debate remains about the precise role and importance of online activities in relation to understanding youth suicide risk.

To inform and advance public health programmatic activities that use technology to prevent suicide, particularly within schools, we performed a matched case-control study. The main objectives of this study were to elucidate whether the occurrence of severe suicide/self-harm alerts among youths are evident from preceding online activities and identify online risk factors associated with suicide/self-harm alerts.

## Methods

Bark’s free online safety tools are currently used in more than 2600 school districts in the US, covering more than 5 million children, making it the largest provider of online student safety tools for school settings.^27^ Because computer use is a key component of contemporary education and testing frameworks, schools now routinely provide computing devices to all students. The online safety tools monitor and send alerts to schools and parents for issues detected on school-provided computers, tablets, and cloud-based accounts for content threatening to the health and well-being of students, such as messages about self-harm, suicidal ideation, online predators, bullying, or threats of violence. A wide variety of content is evaluated, including text, images, and video from activities such as email and web browsing in both English and Spanish. Content is able to be monitored broadly across school technology, which consists of Google G Suite software including email (Gmail), web browsing (Google Chrome), and document storage and messaging (Google Drive). No data from personal (nonschool provided) devices are included. Examples of types of data available include an email sent to a friend using a school email account, a text messaging conversation conducted over Google Drive, and a Google search conducted on a school-provided tablet or computer. Schools are enrolled by school administrators and a school's use of the online safety tool is dependent on the school having obtained parent/guardian permission. School systems participating in the online monitoring platform report the following characteristics: location type (11% city, 42% rural, 23% suburban, and 24% town), 15.1:1 student:teacher ratio, an average of 2986 (52%) male, 66% White race, 2.5% charter school, and an average of 5742 students in each district.

This project uses only anonymous retrospective information from implementation of the online monitoring platform’s programs and services, and all analyses were conducted securely within the online safety tool by its authors. The project was conducted as part of work to improve suicide prevention programs, and this retrospective observational analysis of secondary, administrative data was reviewed and approved by the online safety tool company’s internal review process and consistent with its terms of service and data use policy. The online safety tool provides the ability for any individual to opt out of services and data collection. The project was also reviewed by the Centers for Disease Control and Prevention and deemed exempt from institutional review board review as secondary data analysis. This study followed the Strengthening the Reporting of Observational Studies in Epidemiology (STROBE) reporting guideline for case-control studies.

The study used secondary deidentified administrative data collected by the online safety tool through its software installed on school district devices from July 27, 2019, to May 26, 2020. The analysis used a retrospective, matched case-control design with a 1:5 match. Cases for inclusion were those that the online safety tool identified as having 1 or more severe suicide/self-harm alerts requiring notification of school administrators; severe suicide alerts are statements by youths indicating imminent or recent suicide attempts and/or self-harm and are identified by natural language processing–based machine learning models developed by the online safety tool (eTable in the Supplement provides examples). All alerts were reviewed by a trained team of reviewers to confirm their validity and ensure that the suicide attempt/self-harm behavior was related to the first-party user and not, for example, a conversant or third party.

Each case was matched to 5 controls on location (enrolled in the same school), the amount of observation time available (first monitored on the same day), amount of email volume (sent ±25% the amount of emails), and activity on Google Drive and Google Chrome. Matching on age and sex was not performed so that their associations could be examined in regression models.

To evaluate the association of various explanatory variables with the outcome of suicide/self-harm alerts, we examined multiple potential risk factors for youth suicide (eTable in the Supplement). These variables included alerts for 8 types of abuse-related behavior identified by the online monitoring platform: cyberbullying, drug-related content, sexual content, violence, hate speech, profanity, depression, and low-severity suicide/self-harm alerts (third-party content viewed but not sent by the user that is related to suicide or self-harm). These factors represent a combination of variables with theoretical and literature-based underpinnings,^28^ as well as variables that have been identified by parents and school systems as of interest. All of the potential risk factors examined had been detected via the online monitoring platform abuse-detection models before the severe suicide alert being studied as the outcome variable. To detect abuse-related content, the online monitoring platform uses a deep neural network trained on millions of posts reviewed by a team of trained reviewers. Classification accuracy for all alerts is greater than 90%, and accuracy for severe suicide/self-harm alerts is above 95%. We also examined the mean sentiment score of messages/posts, calculated on a −1 to +1 scale using the VADER algorithm, a widely used sentiment-scoring approach tuned specifically to the kind of informal, conversational content in this population.^29^

Because age and sex information were not directly available, age was inferred based on grade level and sex was inferred using a mapping of first names to sexes established through analysis of names in US Census data.^30^ Information on race and ethnicity was not available. We also identified the number of discrete activities monitored for each account for use as a denominator variable. Activities are individual online actions, such as individual posts, texts, emails, messages, searches, or web page views. Quantifying the total number of activities allows us to calculate the proportion of online activities that are related to a certain alert, such as violent content. This normalization allows us to assess the absolute amount of abusive and nonabusive content as well as relative rates of such content.

### Statistical Analysis

We first performed descriptive analyses, quantifying the proportion of online activities that were related to a given risk factor, such as cyberbullying, stratified by case-control status. Because a precise timestamp exists for all online activities, the risk factors identified all occurred preceding any severe suicide/self-harm alerts in cases or the identical matched time point for controls.

We conducted 3 separate regressions to more fully elucidate the association of preceding online risk factors and subsequent severe suicide/self-harm alerts. First, because pathways between mental health-, violence-, and substance-related risk factors are complex and highly intertwined, we conducted a regression examining each risk factor independently while controlling for age, sex, and the total number of online activities. Results from this regression reveal the total association of each risk factor with subsequent suicide risk through both direct and indirect pathways. Second, to better understand which risk factors have independent, direct associations with subsequent severe suicide/self-harm alerts, we entered all risk factor variables into a multivariable regression model. Third, because earlier research on adverse childhood experiences has revealed that there is a stepped and increasing effect on health, including suicide, with the presence of multiple types of adverse childhood experiences,^31^ we calculated the number of specific categories of online risk factors experienced by each child (0, 1, 2, 3, 4, and ≥5) and examined the odds of severe suicide/self-harm alerts for each level.

For all regressions we used conditional logistic regression to account for the matched case-control design. Each risk factor variable representing the percent of activity that was for a given alert/risk factor was transformed using an empirical logit transformation to place the feature on the same log-odds scale as the outcome.^32^ A model that used raw percentages as independent variables violated the Hosmer-Lemeshow test.^33^ Models also included age, sex, the number of total activities (also log-transformed owing to right skew), and the mean sentiment score as specified in each table. All analyses were conducted in R, version 3.6.2 (R Project for Statistical Computing); statistical significance was defined as 2-tailed *P* < .05.

## Results

Using the inclusion criteria described above, we identified 227 youths (cases) who were subsequently matched to 1135 controls. Overall mean (SD) age was 13.3 (2.41) years; cases and controls did not differ significantly on age (mean [SD], cases: 13.1 [2.18]; controls, 13.3 [2.46] years), sex (227 cases: 118 [52%] female, 109 [48%] male; 1135 controls: 545 [48%] female, 590 [52%] male), or the volume of online emails sent, documents drafted, or web searches conducted (Table 1).

**Table 1.** Demographic Characteristics and Online Activity Patterns for Case and Control Populations, 2019-2020^a^

Group	No.	Age, mean (SD) [range], y^b^	Sex, No. (%)^c^		Activities, mean (SD)^d^		
			Female	Male	Gmail	Google Drive	Google Chrome
Cases	227	13.1 (2.18) [7-18]	118 (52)	109 (48)	1270.98 (3559.77)	133.43 (165.10)	1448.10 (2899.59)
Controls	1135	13.3 (2.46) [6-18]	545 (48)	590 (52)	1027.72 (3220.83)	146.90 (383.04)	1125.72 (2723.44)

^a^ Data from implementation of Bark’s programs and services.

^b^ Age imputed from grade.

^c^ Sex imputed from name through analysis of names in US Census data.^30^

^d^ Activities refer to the number of discrete online actions, such as sending an email (through Gmail), sharing a message in Google Docs (in the case of Google Drive), or conducting a web search (in the case of Google Chrome).

The Figure presents descriptive information on the percentage of all online activities related to a given risk factor among cases and controls. Of the 8 potential risk factors examined, all except hate speech demonstrated significant differences in prevalence between case and control populations. Cyberbullying was the most prevalent risk factor, comprising 1.97% of all online activities among cases and 0.96% among controls. Violence-related content was also prevalent, comprising 0.86% of online activities among cases and 0.40% among controls.

**Figure.**
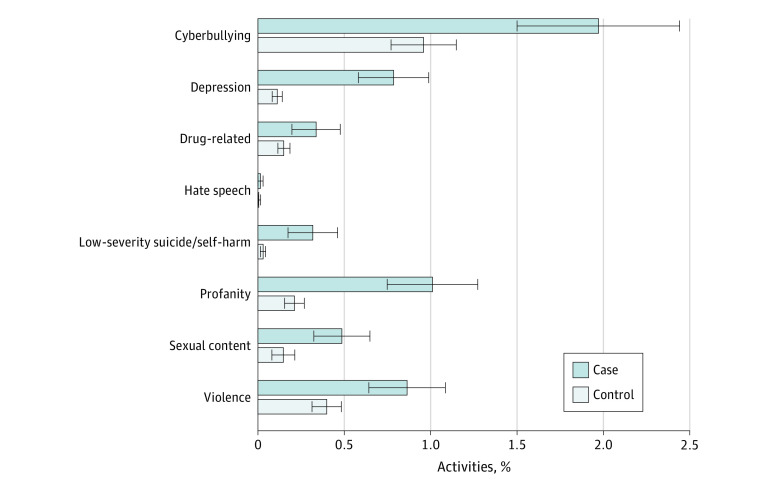
Percentage of Online Activities Related to a Given Risk Factor Among Cases and Controls, 2019-2020 Error bars represent 95% CIs.

Table 2 reports the total association of each risk factor with the subsequent occurrence of a severe suicide/self-harm alert while controlling for the amount of online activities, age, sex, and sentiment score. Although each of the risk factors examined exhibited an association with the severe suicide/self-harm alerts, depression-related content had the largest association with subsequent severe suicide/self-harm alerts (adjusted odds ratio [aOR], 1.82; 95% CI, 1.63-2.03).

**Table 2.** Associations Between Online Risk Factors and Subsequent Severe Suicide/Self-harm Alert, 2019-2020^a^

Risk factor	aOR (95% CI)	*P* value
Profanity	1.70 (1.55-1.88)	<.001
Cyberbullying	1.49 (1.37-1.62)	<.001
Depression	1.82 (1.63-2.03)	<.001
Low-severity suicide/self-harm	1.76 (1.57-1.99)	<.001
Violence	1.39 (1.28-1.51)	<.001
Drug-related	1.17 (1.09-1.26)	<.001
Sexual content	1.50 (1.36-1.65)	<.001
Hate speech	1.22 (1.01-1.47)	.04

Abbreviation: aOR, adjusted odds ratio.

^a^ Models examined each risk factor separately while adjusting for age, sex, and total volume of online activities. Models did not block mediators (eg, the association of cyberbullying with suicide by increasing depression), and thereby show total direct and indirect associations of each potential risk factor with suicide risk.

To further understand the association of each risk factor with subsequent severe suicide/self-harm alerts, Table 3 presents results from a multivariable regression model controlling for each of the other risk factors, thereby blocking any mediating or indirect pathways between a given risk factor and the outcome variable. Results from this model revealed associations for depression (aOR, 1.39; 95% CI, 1.18-1.64), profanity (aOR, 1.35; 95% CI, 1.20-1.53), and sexual content (aOR, 1.19; 95% CI, 1.05-1.35).

**Table 3.** Online Risk Factors With Direct Associations on Severe Suicide/Self-harm Alert, 2019-2020^a^

Independent variable	aOR (95% CI)	*P* value
Age	0.93 (0.83-1.04)	.19
Sex, male	1.15 (0.80-1.66)	.45
Total volume of online activities	1.40 (1.03-1.92)	.03
Sentiment score	0.82 (0.27-2.52)	.73
Risk factors		
Profanity	1.35 (1.20-1.53)	<.001
Cyberbullying	1.12 (0.99-1.26)	.06
Depression	1.39 (1.18-1.64)	<.001
Low-severity suicide/self-harm	1.13 (0.94-1.34)	.19
Violence	1.05 (0.93-1.18)	.41
Drug related	0.96 (0.87-1.06)	.45
Sexual content	1.19 (1.05-1.35)	.01
Hate speech	1.02 (0.79-1.30)	.89

Abbreviation: aOR, adjusted odds ratio.

^a^ Results are from a multivariable model that includes all terms shown in table to block all pathways except direct associations between potential explanatory variables and the outcome of a severe suicide alert. Total volume of online activities was log-transformed and online risk factor variables logit-transformed to normalize data.

In addition, Table 4 presents associations between the total or cumulative number of risk factor categories and the subsequent occurrence of severe suicide/self-harm alert risk. There was an intensifying risk of experiencing a severe suicide/self-harm alert with the number of categories of risk factors that a youth displayed. For example, youths with 5 or more of the 8 risk factors we examined had a markedly increased risk of a subsequent severe suicide/self-harm alert (aOR, 78.64; 95% CI, 34.39-179.84; *P < *.001).

**Table 4.** Cumulative Risk of Severe Suicide/Self-harm Alert Associated With Presence of Multiple Online Risk Factors^a^

Risk factor	aOR (95% CI)	*P* value
Age	0.94 (0.85-1.04)	.24
Sex		
Female	1 [Reference]	
Male	1.03 (0.73-1.44)	.88
Total volume of online activities	1.15 (0.86-1.53)	.36
Sentiment score	0.55 (0.19-1.60)	.27
Total No. of risk factors present		
0	1 [Reference]	
1	4.19 (1.97-8.92)	<.001
2	5.55 (2.45-12.50)	<.001
3	15.24 (6.79-34.21)	<.001
4	34.34 (14.75-79.96)	<.001
≥5	78.64 (34.39-179.84)	<.001

Abbreviation: aOR, adjusted odds ratio.

^a^ Results shown are from a single multivariable model that controlled for all terms shown in the table.

## Discussion

To our knowledge, this study is the first longitudinal examination of online risk markers for youth suicide-related behavior that (1) allows for examination of many diverse types of online risk behaviors beyond overall screen time, (2) uses observed rather than self-reported online activity, and (3) possesses detailed temporal information on risk factors to strengthen study of their association with the outcome of interest. The findings support the importance of understanding and preventing exposure to harmful online activities among youths as a component of youth suicide prevention strategies.

Analyses examining total (direct and indirect) associations revealed that all of the online risk factors exhibited an association with subsequently having a severe suicide/self-harm alert, with the possible exception of hate speech, given that multiple comparisons where made. Variables with direct associations with severe suicide/self-harm alerts included depression, profanity, and sexual content. The association between profanity and severe suicide/self-harm alerts could be a result of profanity serving as a proxy for challenges in emotional regulation as a consequence of mental health deterioration or simply as a general proxy for life stressors not captured in the other variables examined. Perhaps the greatest signal for increased risk was noted when examining the cumulative presence of different types of risk factors in a youth’s life.^34^

Depression-related alerts had the largest association with subsequent severe suicide/self-harm alerts. This finding is consistent with literature identifying depression as the leading risk factor for suicide,^35^ lending support to the internal and construct validity of the online data used.

Exposure to cyberbullying content was the most prevalent online alert and also was associated with severe suicide/self-harm alerts. This finding is also supported by literature from both survey and clinical data documenting the harmful effect of bullying on youth mental health.^36^

Results from regression modeling reported in Table 3 reveal that the associations of many online risk factors flow through or are mediated by other risk factors. This finding was expected, as the interrelatedness of risk factors for suicide, violence, and substance use are well recognized.^5,37^ For example, although a cyberbullying episode on a given day may prompt a youth to impulsively attempt suicide (a direct response), most of the effect of cyberbullying exposure may be mediated through increased levels of depression over time as a result of persistent cyberbullying.

The results exploring the cumulative association of multiple types of risk factors are consistent with the literature on adverse childhood experiences.^31,38^ Specifically, there is an increasing risk of severe suicide/self-harm alerts with each additional type of risk factor. Thus, the cumulative effect of multiple risk factors can be observed from passive online date.

Improved early identification of individuals at risk of suicide has been a long-standing challenge and is a major focus of ongoing suicide prevention research.^39,40,41,42^ Our findings suggest novel avenues for more timely and efficient assistance and youth suicide prevention efforts. Although there are important ethical and privacy considerations when using online, digital, or linked data, efforts to improve mental health using passive digital information or other administrative data are being researched, tested, and used.^43,44,45^ Conducted carefully and ethically, such approaches have the potential to help prevent devastating outcomes for families, such as youth suicide.^46^

### Limitations

This study has limitations. First, the behaviors we examined are focused on online environments. Although each of the youths exhibited severe suicide/self-harm alerts and warranted notification of parents and schools for immediate aid, we do not have access to objective health end points, such as hospitalizations. Nonetheless, we are aware of no work to date that links health care system data on youth suicide to online records from youths. Furthermore, because our study was observational, we cannot make causal claims about the association between online activities and suicide. Second, the precise definition of what constitutes an online risk is subject to debate. Future research with a larger sample size can work to better understand differences between the severity level for each risk factor. However, our online risk factors, as currently constructed, were associated with our outcome of interest. An additional area for future work also includes better characterizing age differences; most study participants were approximately middle-school aged and understanding how risk changes with age is important.

A small portion of the period studied occurred during the emergence of COVID-19 in the US when schools were shifting to increased digital instruction. The study was underpowered to fully explore differences between cases during this period and non–COVID-19 periods; however, a transition to increased use of school computing devices would theoretically increase our ability to capture abuse-related behaviors. In addition, we were unable to fully assess all online activities, such as those conducted on personal devices or not connected to school accounts.

## Conclusions

This research presents efforts at better understanding the association between a variety of potential risk behaviors and subsequent online suicide/self-harm behavior. Parents, clinicians, and suicide prevention organizations are increasingly faced with questions about the role and importance of online behaviors to suicide. This study provides information in this area to guide future research and suicide prevention activities.

## Notes

Supplement. **eTable.** Type and Definition of Online Risk Factors for Suicide/Self-Harm Examined
