# Supplementary material for: Association of Online Risk Factors With Subsequent Youth Suicide-Related Behaviors in the US
Source: JAMA Netw Open. 2021 Sep 20;4(9):e2125860. doi: 10.1001/jamanetworkopen.2021.25860 (PMC8453319; doi:10.1001/jamanetworkopen.2021.25860)
Supplement: Supplement. — eTable. Type and Definition of Online Risk Factors for Suicide/Self-Harm Examined [file jamanetwopen-e2125860-s001.pdf]

Supplementary Online Content

Sumner SA, Ferguson B, Bason B, et al. Association of online risk factors with subsequent youth suicide-related behaviors in the US. *JAMA Netw Open*. 2021;4(9):e2125860. doi:10.1001/jamanetworkopen.2021.25860

eTable. Type and Definition of Online Risk Factors for Suicide/Self-Harm Examined

This supplementary material has been provided by the authors to give readers additional information about their work.

© 2021 Sumner SA et al. *JAMA Network Open.*

eTable 1. Type and Definition of Online Risk Factors for Suicide/Self-Harm Examined

**Variable**	**Definition**	**Example Text**
**Outcome**		
Severe Suicide/Self-Harm	Statements indicating imminent or recent suicide attempts and/or self-harm by the user	*Bye, I'm not sure you'll get this but I'm going to commit suicide today. I love you, thank you for trying to help me but I can't hang on anymore.*
**Potential Risk Factors**		
Cyberbullying	Name-calling, gossiping, and other mean-spirited comments; hateful and/or threatening messages; can be user generated or exposure to third-party content	*You're such a loser.*
Sexual Content	Sexually suggestive language or visual media; media or graphic descriptions depicting sexual acts or abuse; can be user generated or exposure to third-party content	*Send a nude pic.*
Depression	User generated content classified as showing signs of ongoing negative self-esteem, intense and prolonged feelings of hopelessness and worthlessness, and suicidal ideation without stated plan	*I can't do anything right anymore.*
Low-Severity Suicide/Self-Harm	Third-party content (viewed but not sent by the user) related to suicide or self-harm	*It's so hard for me to go on like this. (when statement is third party content made by another and viewed by user)*
Drug-Related Content	User discussion of consuming drugs and/or alcohol or viewing of third-party content related to drugs/alcohol	*Bring the weed to my house.*
Violence	User generated content or viewing of third-party content related to violence such as media depicting violence and/or threats of violence	*If I see you again, I'm going to kill you.*
Hate Speech	Discriminatory jokes or memes; derogatory comments about race, gender, or other protected classes; comments are directed at a group rather than an individual	*I hate Chinese people.*
Profanity	Use of one or more words generally deemed as profane.	*F*ck off*

Note: All examples edited for anonymity.

© 2021 Sumner SA et al. *JAMA Network Open.*
